# Clinical pharmacy practice in the care of Chronic Kidney Disease patients: a systematic review

**DOI:** 10.1007/s11096-019-00816-4

**Published:** 2019-04-09

**Authors:** Fatma Al Raiisi, Derek Stewart, Fernando Fernandez-Llimos, Teresa M. Salgado, Moustafa Fahmy Mohamed, Scott Cunningham

**Affiliations:** 1 School of Pharmacy & Life Sciences, Robert Gordon University, Aberdeen, Scotland, UK; 2 Research Institute for Medicines and Pharmaceutical Sciences (iMed.UL), Faculty of Pharmacy, University of Lisbon, Lisbon, Portugal; 3 Department of Pharmacotherapy & Outcomes Science, Center for Pharmacy Practice Innovation, Virginia Commonwealth University School of Pharmacy, Richmond, VA USA; 4 Oman Pharmacy Institute, Ministry of Health, Muscat, Oman

**Keywords:** Chronic kidney disease, Clinical pharmacy, Pharmacist, Systematic review

## Abstract

*Background* Clinical pharmacy services have potential to contribute significantly to the multidisciplinary team providing safe, effective and economic care for patients. Given recent practice developments (e.g. polypharmacy reviews and pharmacist prescribing) there is a need to provide a current synthesis of the evidence base for characteristics and outcomes of clinical pharmacy practice in chronic kidney disease patients. *Aim of the review* To critically appraise, synthesise and present the available evidence of the characteristics (structures and processes) and outcomes of clinical pharmacy practice as part of the multidisciplinary care of patients with chronic kidney disease. *Method* PubMed, International Pharmaceutical Abstracts (IPA), Cumulative Index to Nursing and Allied Health Literature (CINAHL), Medline and Scopus were searched for peer reviewed papers using improved search strategy. Included studies were quality assessed using Downs and Black tool for controlled studies and the mixed methods appraisal tool for all controlled and non-controlled studies. Data were extracted and synthesised using a narrative approach. Screening, quality assessment and data extraction were performed by two independent researchers. Ethics approval was not required. *Results* Forty-seven studies were identified from a variety of countries, with 31 based in a hospital setting. Controlled study designs were employed in 20, with only ten of these using randomisation. Resources available for service provision were poorly reported in all papers. Positive impact on clinical outcomes included significant improvement in parathyroid hormone, blood pressure, haemoglobin and creatinine clearance. Pharmacists identified 5302 drug related problems in 2933 patients and made 3160 recommendations with acceptance rates up to 95%. Impact on humanistic outcomes was shown through improvement in health related quality of life and patient satisfaction. Economic benefits arose from significant cost savings through pharmaceutical care provision. *Conclusion* While there is some evidence of positive impact on clinical, humanistic and economic outcomes, this evidence is generally of low quality and insufficient volume. While the existing evidence is in favour of pharmacists’ involvement in the multidisciplinary team providing care to patients with chronic kidney disease, more high-quality research is warranted.

**Electronic supplementary material:**

The online version of this article (10.1007/s11096-019-00816-4) contains supplementary material, which is available to authorized users.

## Impacts on practice

- Understanding fully the structures, processes and relevant outcomes associated with clinical roles of pharmacists is essential to make best use of resource for optimal patient care.
- There has been a significant volume of research of the clinical role of pharmacists in Chronic Kidney Disease, but it is of limited detail and quality.
- There is a need for agreed standard sets of outcomes for clinical pharmacy practice and research in chronic kidney disease.

## Introduction

Chronic Kidney Disease (CKD) continues to be a global concern with a high risk of mortality, frequent hospitalisation and reduced life expectancy [1]. Most patients have co-morbid conditions such as cardiovascular and mineral bone diseases [2]. Clinical pharmacy services have the potential to contribute significantly to the multidisciplinary team providing safe, effective and economic care [3]. Key clinical pharmacy roles in the multidisciplinary care of CKD patients were described by two renal pharmacy consultants Mason and Bakus in 2010 [4]. These roles included specific areas such as managing anaemia, renal mineral bone disease and hypertension, as well as more general medicines selection and review [4]. Another major role pharmacists can play is to contribute to renal drug cost management [5]. An emerging role is the potential for the pharmacist to prescribe and modify medicines, which has now been implemented into practice in the United Kingdom (UK), United States (USA) and New Zealand [6]. There is a need to establish the evidence base of the impact of clinical pharmacy in the care of CKD patients. In 2012, Salgado et al. published a systematic review which included synthesis of the peer reviewed literature up to March 2010 [7]. The original review identified 37 studies (38 articles), involving 4743 participants. Majority of the papers were of uncontrolled design (80%) [7]. Twenty-one articles (55.3%) reported outcome measures and process indicators, 4 (10.5%) reported only outcome measures, thirteen (34.2%) reported only process indicators and none reported structures [7]. Pharmacists identified 2683 drug-related problems in 1209 patients. The results from controlled studies (average quality score 0.57, SD = 0.10) reported that pharmacists’ interventions reduced all-cause hospitalisations, reduced the incidence of end-stage renal disease or death in patients with diabetic nephropathy, improved management of anaemia, blood pressure, calcium and phosphate parameters and lipid management [7]. The uncontrolled studies included in the original review shown positive impact of pharmacists’ interventions on the reduction of transplant rejections and fewer adverse events [7]. The reviews main limitations were selection and language bias which might affect the quality of the systematic review. Salgado et al. concluded that the evidence of pharmacists’ interventions in patients with CKD is scarce, of variable quality and with heterogeneous outcomes [7]. Since the publication of the original review by Salgado et al., the prescribing practice has continually developed with new services and models of care being developed and embedded into clinical pharmacy practice. Hence, there is a need to update and extend the review. Given developments in clinical pharmacy globally, it is likely that further research has been reported thus an up-to-date synthesis is warranted.

## Aim of the review

The aim of this review was to critically appraise, synthesise and present the available evidence for the structures, processes and related outcomes of clinical pharmacy practice as part of the multidisciplinary care of patients with CKD. The specific review questions were:

- What clinical pharmacy practice related resources (structures, e.g. the multidisciplinary team, clinical pharmacy skill mix and time allocation) are in place and how are these matched to healthcare needs and demands to enable provision of care to chronic kidney disease (CKD) patients?
- What activities are performed (processes, e.g. medication review, prescribing) to care for patients with CKD, how and when are they performed?
- What are the outcomes of the structure and the processes on the effectiveness (Economic, Clinical, and Humanistic Outcomes (ECHO) model) [8] of care provided?

## Method

### Data sources

The systematic review protocol was registered with the International Prospective Register of Systematic Reviews (PROSPERO) (PROSPERO 2017 CRD42017065258). The protocol was constructed in accordance with PRISMA-P (Preferred Reporting Items for Systematic review and Meta-Analysis Protocols) standards [9], and the review conducted and reported in accordance with PRISMA (Preferred Reporting Items for Systematic Review and Meta-Analysis) standards [10].

The Cochrane database was searched to identify any relevant systematic reviews. An electronic search of relevant databases (PubMed, International Pharmaceutical Abstracts (IPA), Cumulative Index to Nursing and Allied Health Literature (CINAHL), Medline and Scopus) was conducted from March 2010 to December 2018 thus providing an update on the review of Salgado et al. [7]. The search was carried out using Medical Subject Headings (MeSH) and other appropriate subject headings and text words. Scoping searches were conducted prior to finalising the search strategy. Boolean operators such as truncations (*), wild cards ($), adjacent search options (e.g. adj2) were used where relevant. The following grouped terms were initially searched separately then in combination by two independent reviewers (FA & SC). The primary search was conducted using the improved search strategy of the same terms as the original review as follows:

PubMed, IPA, CINAHL: (“pharmaceutical services” [MH+] OR “pharmacy” [MH+] OR “Pharmacies” [MH] OR “Pharmacists” [MH] OR “clinical pharmacist*” [TI/AB/SU] OR “clinical pharmacy” [TI/AB/SU] OR “clinical pharmacies” [TI/AB/SU] OR “pharmacist*” [TI/AB/SU] OR “pharmaceutical services” [TI/AB/SU] OR “pharmacies” [TI/AB/SU] OR “pharmacy” [TI/AB/SU]) AND (“kidney diseases” [MH+] OR “renal replacement therapy” [MH+] OR “proteinuria” [MH+] OR “CKD” [TI/AB/SU] OR “nephropathy” [TI/AB/SU]).

Scopus:

(“Pharmaceutical care” [TI/ABS/KEY] OR “Pharmacist” [TI/ABS/KEY] OR “Clinical pharmacy” [TI/ABS/KEY]) AND (“Chronic Kidney Disease” [TI/ABS/KEY] OR “Renal replacement Therapy” [TI/ABS/KEY] OR “Haemodialysis” [TI/ABS/KEY] OR “Kidney failure” [TI/ABS/KEY]). The bibliography list of included studies was reviewed to further identify additional references.

### Study selection and data extraction

Only quantitative studies (randomised and non-randomised controlled and uncontrolled trials, cohort studies and before and after evaluations) published in peer-reviewed journals were included in the review. Papers published in English and focusing on researching clinical pharmacy practice and the role of the pharmacist in managing patients with CKD were included. Studies not addressing the topic, literature based only on conceptual models, i.e. lacking empirical evidence, grey literature including conference proceedings, abstracts and unpublished studies were excluded. Observational studies were excluded since they did not address the aim of this review.

Title and abstract screening and quality assessment for inclusion were conducted independently by two reviewers (FA and SC), with any disagreements resolved by discussion with a third independent reviewer (DS).

### Quality assessment

An independent, duplicate quality assessment of each study was undertaken (DS, TJ, FA & SC). All controlled, uncontrolled and descriptive studies were assessed using the mixed methods appraisal tool (MMAT), a validated and unique tool for appraising different types of study designs [11]. All controlled studies included in this review were additionally assessed for quality using the Downs and Black’s method in line with the original review [12], a validated tool with a scoring scale consisting of 27 questions grouped into five domains (reporting, external validity, bias, confounding and power). The total score is 32 and is expressed as rates, the higher the score the better the quality of the paper in terms of methodology (maximum is 1) [12]. To classify scores, the approach of Machado et al. was applied [13] (i.e. < 0.5 was considered ‘weak’, 0.5–0.69 were ‘fair’, 0.7–0.79 ‘good’ and 0.8–1.0 ‘very good’).

### Data extraction

Data extracted included: primary author, year of publication, aim/objectives, design, duration, setting, participants, pharmacist interventions, key findings or main outcomes and conclusion. Structures, processes and outcomes were adapted from Donabedian’s quality of care model [14]. Structure was defined as the ‘resources required for the pharmacist to be able to provide care to renal patients such as requiring special training, availability of policies and procedures for practice etc’. Process was defined as ‘the activities that are performed by the pharmacist on a daily basis or on specific intervals and how and when they are performed. These activities may include: daily clinical rounds, involvement in patients’ management plans, medication reviews, therapeutic recommendations and pharmacist prescribing. Outcome measures included clinical outcomes such as: clinical parameters, medication-related adverse events, mortality and morbidities, humanistic outcomes such as: quality of life and economic outcomes such as: rate of hospitalisation and cost of inappropriate therapies. In addition, pharmacists’ intervention was defined in the previous review as “any action with the aim of modifying the process of use of drugs, either in patients’ activities or in medical or health care practitioners’ activities” [7].

### Data synthesis

Due to heterogeneity in the data obtained from the included papers (type of patients, study design, outcomes measured), only descriptive and narrative synthesis was possible. All findings were considered by two independent reviewers to ensure robustness and consistency in execution of the review process.

## Results

### Study selection and data extraction

No systematic reviews were identified from the Cochrane database and no additional primary studies were identified from the bibliography lists of included studies.

**Fig. 1:**
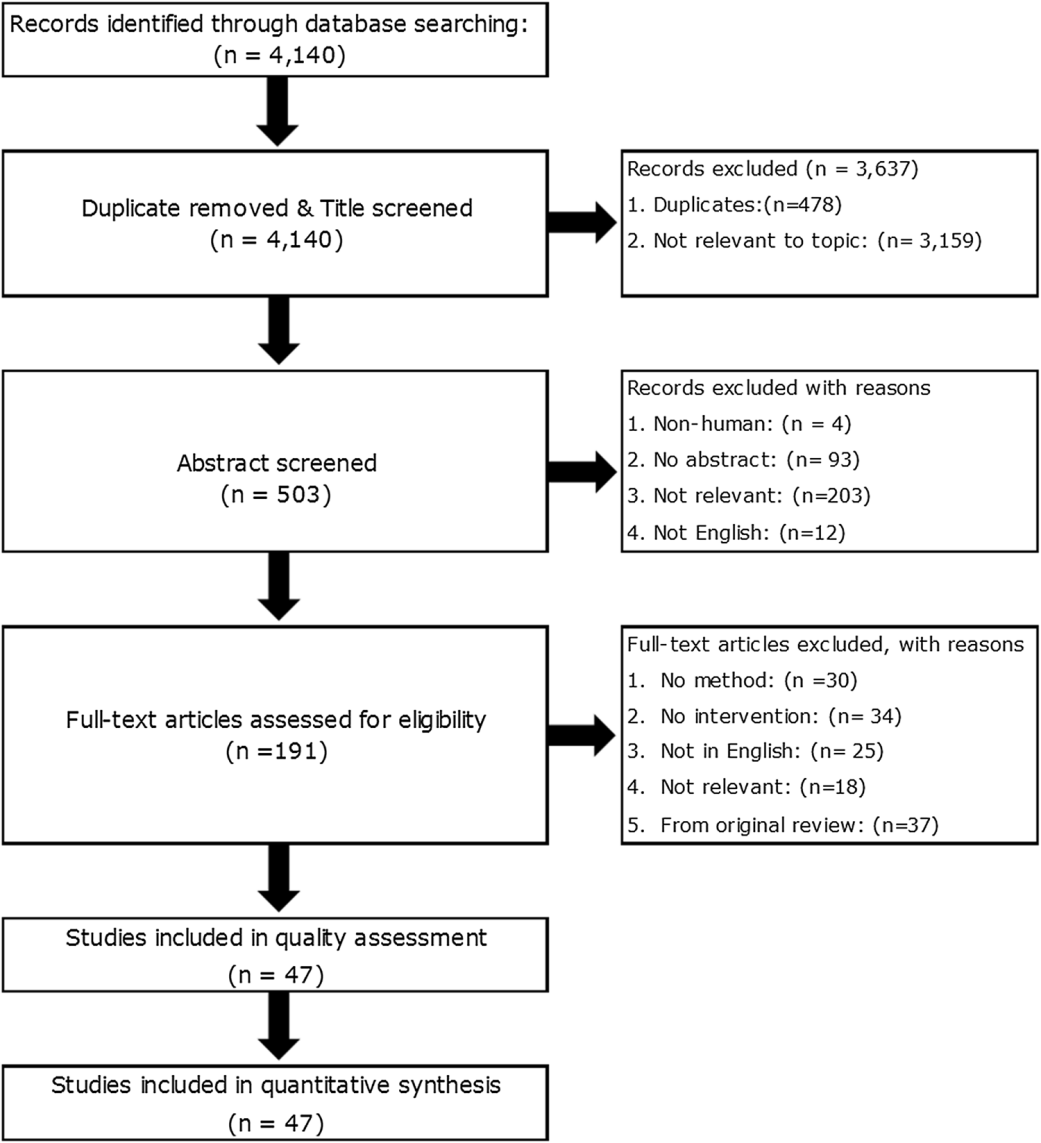
PRISMA Chart describing study retrieval and selection

Databases searches identified 4140 potential articles to screen further for eligibility (Fig. 1). Only 47 articles met the inclusion criteria and after quality assessment were of a standard deemed acceptable for inclusion in the review.

### Quality assessment

**Fig. 2:**
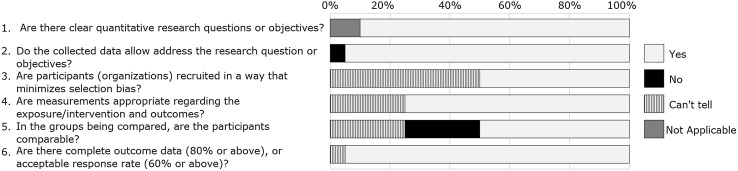
Stacked bar chart representing quality of quantitative Randomized Controlled Trials (n = 10). The % values above represents the proportion for each response as agreed between reviewers for the papers included for each study design

**Fig. 3:**
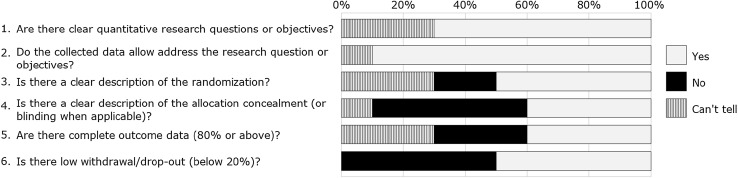
Stacked bar chart representing quality of quantitative non-randomized studies (n = 20). The % values above represents the proportion for each response as agreed between reviewers for the papers included for each study design

**Fig. 4:**
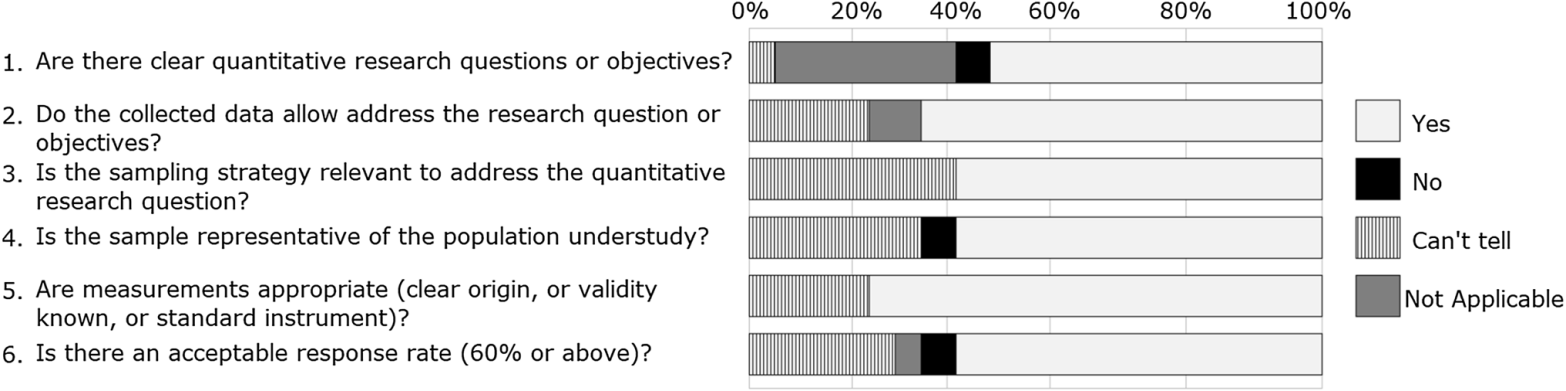
Stacked bar chart representing quality of quantitative descriptive studies (n = 17). The % values above represents the proportion for each response as agreed between reviewers for the papers included for each study design

The Downs and Black’s mean score of the 20 controlled studies was 0.557 (SD = 0.075). All papers presented ‘fair’ quality with the exception of four that scored < 0.5 and was therefore considered ‘weak’ quality. The quality assessment of all the included studies using the MMAT tool for the randomised (n = 10), non-randomised (n = 20) and descriptive studies (n = 17) are shown in Figs. 2, 3 and 4.

### Data extraction

Tables 1 and 2 detail the data extraction characteristics of controlled and uncontrolled studies included in the systematic review [15–61].

**Table 1:** Characteristics of controlled studies included in the systematic review

Study year Country	Study design (duration)	Study setting	Aim	Participants		Intervention	Control	Main clinical outcomes achieved
				N (at baseline)	Age (years), mean (SD)			
Santschi et al. (2011) Canada [48]	Cluster, randomised study (6 months)	Primary Care, Community Pharmacies. Multidisciplinary pre-dialysis clinic	To evaluates the impact of ProFiL on BP control and management of hypertension treatment	90 CKD patients	ProFiL group 71.9 (10.4), and usual care group 73.3 (7.7)	(1) A 3-h training workshop for community pharmacists (2) A communication network to facilitate the transfer of clinical information between the pre-dialysis clinic and community pharmacists (3) A pharmaceutical consultation service by hospital pharmacists with expertise in nephrology (n = 48)	Usual care (n = 41)	Adjusted mean BP changes, were (− 6.9/− 0.4 mmHg in ProFiL patients) compared with (+ 4.7/+ 2.2 mmHg in UC) (between groups differences, *p* value = 0.021/0.348). At 6 months, 44% of ProFiL and 24% of UC patients achieved their BP targets. Patients with written hypertension recommendations had a greater decrease in mean systolic BP (− 11.6 mmHg; *p* value = 0.035), and BP was controlled in a higher proportion of them (relative risk, 2.14; *p* value = 0.011)
Aspinall et al. (2012) USA [39]	Non-randomised controlled study (6 months)	Primary care setting, Medical centers	To compare the quality of ESA prescribing and monitoring for patients with NDD-CKD in Veterans Affairs Medical Centers with and without pharmacist-managed ESA clinics	572 NDD-CKD patients	Pharmacist-Managed ESA Clinic 73.9 (10.9), Usual-Care 78.4 (8.8), Usual Care at ESA Clinic 76.2 (12.0)	Dosing and monitoring ESA therapy by pharmacists (n = 314) Usual care at ESA clinic site (n = 91)	Usual care (n = 167)	More haemoglobin values were in the target range in pharmacist-managed ESA clinics (71.1% vs. 56.9% for usual-care sites; P < 0.001) Veterans in pharmacist-managed ESA clinics had more haemoglobin measurements on average (5.8 vs. 3.6 in usual-care sites and 3.8 in usual care at ESA clinic sites; *p* = 0.007).
Dashti-Khavidaki et al. (2013) Iran [30]	Cluster, randomised study (12 months)	Haemodialysis ward of a university affiliated tertiary hospital	To assess the impact of pharmaceutical care on HRQoL of haemodialysis patients	92 HD patients	Intervention 55.4 (15.7), control 48.6 (14.7)	Receive clinical pharmacist-led pharmaceutical care in addition to the standard care of the ward as the case group (n = 26)	Control group (n = 34)	Not reported
Via-Sosa et al. (2013) Spain [18]	Non-randomised controlled study (9 months)	Community pharmacies	To evaluate the effectiveness of the community pharmacist intervention in addressing the problem of dosing inadequacy as a consequence of renal impairment in patients over 65 years that were taking 3 or more drugs when compared with usual care	40 community pharmacies 354 CKD patients	Intervention 80.8 (7.3), control 82.9 (7.1)	Pharmacists used a questionnaire to write a report to GPs detailing the DRPs detected and suggesting changes in therapy. GPs to provide written reply to the pharmacists within 14 days (n = 178)	Control group (n = 176)	The difference in the prevalence of dosing inadequacy between the control and intervention group before the pharmacists’ intervention was 0.73% [95% CI (− 6.0)–7.5] and after the pharmacists’ intervention it was 13.5% [95% CI 8.0–19.5] (*p* < 0.001) while the difference in the mean of drug-related problems per patient before the pharmacists’ intervention was 0.05 [95% CI(− 0.2)–0.3] and following the intervention it was 0.5 [95% CI 0.3–0.7] (*p* < 0.001).
Cabello-Muriel et al. (2014) Spain [36]	Non-randomised controlled study (Unclear)	Internal medicine department of a referral hospital	To demonstrate that the intervention of a pharmacist in a monitoring program for patients with CKD improves the outcome of renal function in these patients	249 CKD patients	Intervention 82.4 (7.4), Control 81.2 (8.5)	Pharmacist intervention including patient interview, medication history taking, identification of inappropriate doses of nephrotoxic drugs, daily check of laboratory parameters and proposing dose adjustments to physicians (n = 124)	Control group (n = 125)	Significant differences were noted when comparing CrCl between discharge and admission in both the control and intervention groups (5.1 ± 0.9 vs. 6.4 ± 1.0 *p* < 0.01). The rate of acceptance of the pharmacists’ recommendations was 74%
Debenito et al. (2014) USA [44]	Non-randomised controlled study (6 months)	Primary care setting, health care system	To assess adherence to monitoring guidelines, along with efficacy and safety outcomes, and to quantify medication utilization expenditures among patients using ESA therapy managed by a clinical pharmacy service compared with usual care	101 CKD patients (pre-dialysis)	Intervention 65.6 (14.1), UC 72 (13.3)	Clinical pharmacy services provided to patients attending the Clinical Pharmacy Anticoagulation and Anaemia Service (n = 31)	Usual care (n = 70)	Time to achievement of haemoglobin target was 28 days in the pharmacist-managed group compared with 41 days in the usual care group (*p* = 0.135), while the proportion of patients achieving target haemoglobin was 96.8% compared with 95.7%, respectively (*p* = 0.654). Patients in the pharmacist-managed group used less ESA during the 6-month period, leading to an annualized savings of 1288 USD per patient in drug expenditures
Jiang et al. (2014a) China [35]	Non-randomised controlled study (12 months)	University affiliated tertiary hospital	To describe the development and implementation of pharmacist dosing adjustment for critically ill patients receiving CRRT and to examine the effectiveness of pharmacist interventions	209 patients on CRRT	Intervention58.9 (17.3), No-intervention 61.3 (16.9)	The pharmacists assessed the patients receiving CRRT daily during ICU rounds, and then made dosage adjustment interventions when needed (n = 106)	No-intervention group (n = 103)	Suspected adverse drug events in the intervention group were significantly lower than the pre-intervention group (35 in 27 patients versus 18 in 11 patients, *p* < 0.001). However, there was no significant difference between length of ICU stay and mortality after pharmacist dosing adjustment, which was 8.93 days versus 7.68 days (*p* = 0.26) and 30.10% versus 27.36% (*p* = 0.39), respectively. The majority of identified ADEs caused significant injury (48.6% in the pre-intervention period and 44.4% in the post-intervention period) to the patients involved; the number of these ADEs differed significantly between the two groups (*p* = 0.02).
Jiang et al. (2014b) China [40]	Non-randomised controlled study (12 months)	University affiliated tertiary hospital	To evaluate the effect of clinical pharmacist participation in an ICU team on antimicrobial dosing adjustment intervention for patients receiving CVVH	180 patients on CVVH	Intervention 62.0 (18.4), Control 59.3 (20.6)	Pharmacists assessed critically ill patients receiving CVVH daily during ICU rounds, and made antimicrobial dosage adjustment interventions when needed (n = 93)	Control group (n = 87)	Pharmacists made 256 antimicrobial dosing adjustment recommendations for patients receiving CVVH, of which 224 (87.5%) recommendations were accepted by physicians. In control group, pharmacist dosing adjustment resulted in £1637.7 (2669.5 USD) cost savings per patient, and 2.36 times reduction of antimicrobial-related adverse drug events (ADEs) (11 vs. 26, *p* = 0.002), while length of ICU stay and mortality in ICU showed no significant difference (*p* > 0.05)
Joost et al. 2014) Germany [47]	Non-randomised controlled study (12 months)	Renal transplant unit at a university hospital	To investigate the efficacy of a pharmaceutical care programme for applying adherence management module to enhance kidney transplant patients’ adherence to immunosuppressive medication	74 Tx patients	ICG: 51 (13.3), SCG: 54 (11.9)	Additional pharmaceutical care and counselling provided by the clinical pharmacist after the transplantation Additional meetings with clinical pharmacist at out-patient transplantation care (minimum once per quarter up to maximum of once a month). (n = 35)	Standard care group (n = 39)	Adherence was significantly improved in patients of the ICG (91%) compared with SCG (75%) during the first year after transplantation (*p* = 0.014). Daily adherence measures were already improved within 30–40 days after start of intensified patientcare. Intensified care patients also showed significantly better results for taking adherence (*p* = 0.006), pill count (*p* = 0.008) and drug holidays (*p* = 0.001).
Cooney et al. (2015) USA [15]	Pragmatic, randomised, controlled study (12 months)	Primary care	To evaluate the effect of a pharmacist-based quality improvement program on 1) outcomes for patients with CKD and 2) adherence to CKD guidelines in the primary care setting	2199 CKD patients	Intervention75.5(8.2), control 75.7(8.2)	Phone-based pharmacist intervention, pharmacist-physician collaboration, patient education and a CKD registry (n = 1070)	Usual care (n = 1129)	Improvement in the primary process outcome, measurement of PTH (16.1% in the control arm vs. 46.9% in the intervention arm; *p* < 0.001). Subjects in the intervention arm were prescribed more classes of antihypertensive medications than those in the control arm (*p* = 0.02) Increased % of subjects with a phosphorus and urine albumin to creatinine ratio measured for intervention arm. Satisfaction with the intervention was very positive; 92% of participants
Staino et al. (2015) USA [20]	Non-randomised controlled study (3 months)	Renal transplant clinic at a medical university hospital	To determine if a pharmacist-executed comprehensive chart review could serve as sufficient substitution for direct participation during outpatient clinic visits in the post-discharge follow-up treatment of kidney transplant recipients	219 Tx patients	Intervention 50, comparator 52	Pharmacists provided recommendations via chart review for patients who attended the transplant nephrology clinic. (n = 170)	Comparator group (n = 175)	Not reported
Chang et al. (2016) USA [45]	Pragmatic, cluster, randomised study (18 months)	Primary care	To examine the feasibility of using pharmacist MTM to improve proteinuria screening and CKD management in a large, integrated health system	6 primary care sites, 47 CKD patients	MTM 64.0 (13.2), control 70.6 (9.7)	Pharmacist MTM arm received additional support from the pharmacist at the clinic site These pharmacists received additional education about KDIGO-based screening and management guidelines (n = 24)	Control group (n = 23)	The pharmacist MTM intervention did not significantly improve total proteinuria screening at the population level (OR 2.6, 95% CI: 0.5–14.0; *p* = 0.3). However, it tended to increase screening of previously unscreened patients (78.6% in the pharmacist MTM group compared to 33.3% in the control group; (OR 7.3, 95% CI: 0.96–56.3; *p* = 0.05).
Qudah et al. (2016) Jordan [32]	Randomised controlled study (6 months)	Outpatient haemodialysis units of a university hospital	To evaluate clinical pharmacists role in the management of blood pressure in haemodialysis patients guided by home blood pressure monitoring	60 HD patients	Intervention 55.3 (15.1), and control 51.7 (18.5)	Physician-pharmacist collaborative care to optimize antihypertensive pharmacologic therapy (n = 29)	Control group (n = 27)	46% of patients in the intervention arm achieved BP target (mean home BP ≤ 135/85 mmHg) compared to only 14.3% of patients in the control arm (*p* = 0.02). Average decline in weekly mean home SBP was 10.9 ± 17.7 mmHg in the intervention arm (*p* = 0.004) Weekly mean home systolic blood pressure increased by 3.5 ± 18.4 mmHg in the control arm (*p* = 0.396)
Chia et al. (2017) Singapore [52]	Non-randomised, controlled study (24 months)	Outpatient nephrology clinic of a tertiary hospital	To determine whether a collaborative care (CC) model with pharmacist involvement can reduce admissions and healthcare utilization in patients receiving dialysis, compared to usual care (UC)	134 patients	CC 62 (11.4), UC 60.4 (10.8)	Pharmacists performed medication review, disease and medication counselling. They completed training modules and received 4 sessions of training with an experienced pharmacist before they could provide the service independently	Usual care (n = 190)	CC reduced admissions by 27% (IRR 0.73, 95% CI 0.54–0.99, *p* = 0.047) and shortened mean LOS by 1.3 days [6.7 (2.6) versus. 8.0 (3.2), *p* < 0.001] compared to UC. No significant differences in mortality (*p* = 0.189) or mean healthcare utilization cost (*p* = 0.165) between groups Pharmacists identified 515 DRPs with 429 (83.3%) resolved after review
Mateti et al. (2017) India [53]	Open-label, randomised control study (15 months)	Dialysis centres of teaching (TH), government (GH), and corporate hospitals (CH)	To assess the impact of Pharmaceutical Care (PC) on the HRQoL among HD patients	78 patients	PC group 52.78 (10.45) in TH, 49.15 (12.57) in GH and 52.97 (15.12) in CH. Usual care group 49.40 (12.47) in TH, 48 (17) in GH and 53.77 (11.87) in CH	(1) The PC group received the usual care along with pharmaceutical care delivered by a qualified registered pharmacist. The customized care plan was designed and delivered to the patients on monthly basis based on the condition and need of the patient by the WHO-FIP Pharmaceutical care model. (2) The QoL was assessed using validated KDQoL-36 instrument	Usual care (n = 75)	The HRQoL scores were significantly improved over time in the domains noticed with regard to the “physical functioning, general health, emotional well-being, social functioning, symptom/problem list, and effects of kidney disease” in all the three centres of PC group compared to UC group with *p* < 0.05 The baseline HRQoL score of KDQoL-36 domains such as ESRD-targeted areas were not significantly different in the UC group versus PC group in all the three HD centres The pharmaceutical care provided by a trained pharmacist had positive impact in HRQoL of HD patients
Anderegg et al. (2018) USA [54]	Cluster randomised trial	32 medical offices from 15 states	To determine if hypertensive patients with comorbid DM and CKD receiving a pharmacist intervention had improved BP control and greater reduction in mean BP at 9 months compared with those receiving usual care	227 patients	Intervention group 61.7 (11.6), control 63.1 (12.2)	Pharmacist interviewed patients to review medications, assessed knowledge and then educated the patients on HTN. Individualised care plans were prepared and presented to the physician	108 patients	Intervention group had significantly greater mean systolic blood pressure reduction compared with usual care at 9 months (8.64 mm Hg; 95%, CI − 12.8 to − 4.49, *p* < 0.001). The intervention group had significantly higher BP control at 9 months than usual care (adjusted odds ratio [OR] 1.97, 95%, CI 1.01–3.86, *p* = 0.047 and OR 2.16, 95% CI 1.21–3.85, *p* = 0.0102, respectively)
Mateti et al. (2018 a) India [55]	Open-label, randomised control study (15 months)	Dialysis centres of teaching (TH), government (GH), and corporate hospitals (CH)	To assess the impact of pharmaceutical care on medication adherence, Hb levels, blood pressure (BP), and interdialytic weight gain (IDW) among HD patients	78 patients	As [53]	Tailored care plan has been designed and provided to the PC group patients on monthly basis based on the situation of the patient by the “WHO-FIP Pharmaceutical care model”	Usual care (n = 75)	The PC group had significantly reduced its IDW and BP levels in comparison to UC group at different time intervals with a statistical significance of *p* < 0.05. The Hb levels and medication adherence rate scores of HD patients had significantly increased in PC group compared to UC group at different time intervals
Mateti et al. (2018 b) India [56]	Open-label, randomised control study (12 months)	Dialysis centres of teaching (TH), government (GH), and corporate hospitals (CH)	To assess the cost-effectiveness of pharmaceutical care versus usual care on treatment costs in the patients undergoing maintenance HD	78 patients	As [53]	(1)The pharmacist provided PC to the PC group patients on monthly basis regarding the knowledge about the medications, disease, lifestyle and medication chart review (2) The annual costs of medications, HD, laboratory tests, and travel were collected The economic outcomes were assessed by incremental cost-effectiveness ratio (ICER)	Usual care (n = 75)	The incremental cost-effectiveness ratio for academic, government, and corporate hospitals HD patients of PC group compared with UC group were 86,230 Indian Rupee (INR)/Quality adjusted life year (QALY) ~ (1223.03 USD), 231,016.66 INR/QALY ~ (3276.6 USD), and 87,430 INR/QALY ~ (1240.05 USD), respectively.
Tuttle et al. (2018) USA [57]	Single-blind, randomized, controlled trial (3 months)	Hospital setting and home visits.	To determine the effect of a medication therapy management intervention on acute care utilization after hospitalization in patients with CKD not on dialysis	72 patients	Intervention group 70 (12), control group 69 (10)	A 1- to 2-hour in-home visit from a pharmacist for a medication therapy management (medication review, action plan and list) within 7 days of hospital discharge	69 patients	The primary outcome (composite of hospitalisation/emergency department/urgent care centre visits) occurred in 44% of the intervention group and 41% in control group (*p* = 0.72). Hospital readmission rate was n = 19 (26%) in the intervention group and n = 18 (26%) in the control group (*p* = 0.95). No difference in achievement of goals for BP, haemoglobin, phosphorus, or parathyroid hormone
Xu et al. (2018) Taiwan [58]	Non-randomised, controlled study (12 months)	Kidney transplant clinics of a medical centre.	To evaluate the behavioural and physiological outcomes of pharmaceutical care in kidney transplant recipients	43 Tx patients	RE group 48.6 (8.9). RI group 49.0 (12.8)	The pharmacists provided face-to-face interviews, check-ups for laboratory examinations, and discovery and documentation of DRPs, pharmaceutical consultation, and education	12 Tx patients	Patients in the RE group possessed better knowledge for self-care (49.6 ± 4.8 vs. 38.8 ± 9.1; *p* < .001); however, the differences at 12 months became insignificant (56.4 ± 5.9 vs. 56. ± 4.7; *p* = 0.72) after patients in the IR group had also received routine pharmaceutical care. Besides, serum creatinine level of the RE patients was stable without significant variation (*p* = 0.93), but it demonstrated a rising trend in IR patients (*p* < .01). Patients satisfactory with the intervention was 95.2%

*ADEs* adverse drug effects, *BP* blood pressure, *CI* confidence interval, *CKD* chronic kidney disease, *CrCl* creatinine clearance, *CRRT* continuous renal replacement therapy, *CVVH* Continuous Veno-Venous Hemofiltration, *DRPs* drug related problems, *ESA* Erythropoiesis stimulating agent, *GPs* general practitioners, *HD* haemodialysis, *HRQoL* health-related quality of life, *ICG* intensified care group, *ICU* intensive care unit, *KDIGO* kidney disease: Improving global outcomes, *MTM* medication therapy management, *NDD-CKD* non-dialysis dependant chronic kidney disease, *OR* odds ratio, *PTH* parathyroid hormone, *SBP* systolic blood pressure, *SCG* standard care group, *Tx* transplantation, *UC* usual care

**Table 2:** Characteristics of uncontrolled studies included in the systematic review

Study year Country	Study design (duration)	Study setting	Aim	Participants		Pharmacist interventions	Main clinical outcomes achieved
				N (at baseline)	Age (years), mean (SD)		
Kelly et al. (2008) United Kingdom [43]	Prospective uncontrolled study (18 months)	Diabetes unit of a secondary hospital	To offer stepwise intensive treatment to patients with diabetic nephropathy picked up at the traditional secondary care clinic	116 diabetic nephropathy patients	63.4 (8.6)	Frequent visits to pharmacist led clinic for treatment optimisation, checking of BP, renal function, HbA1c, ACR, FBC, calcium and phosphate. Medical history taking by two sources	Significant improvements in BP (*p* < 0.001), total cholesterol (*p* < 0.001) and HbA1c (*p* < 0.05)
Dashti-Khavidaki et al. (2009) Iran [51]	Prospective uncontrolled study (12 months)	Nephrology and infectious disease wards of a large university hospital	To understand the types of services provided by clinical pharmacists in nephrology and infectious disease wards, the acceptance by physicians and the clinical significance of these services	1105 CKD patients	52.5 (14.1)	Uniform documentation of all clinical pharmacy residents activities and interventions	Not reported
Vessal (2010) Iran [17]	Prospective uncontrolled study (4 months)	Nephrology ward of a university hospital	To determine the impact of a clinical pharmacist on detection and prevention of prescription errors at the nephrology ward of a referral hospital	76 CKD patients	47.7 (17.2)	CP reviewed medication orders and intervention was made after agreement of the attending physician	Although 89.5% of the detected errors caused no harm, 4(4.7%) of the errors increased the need for monitoring, 2 (2.3%) increased length of stay, and 2 (2.3%) led to permanent patient harm
Castelino et al. (2011) India [29]	Prospective uncontrolled study (8 months)	Department of nephrology of a teaching hospital	To explore the potential clinical significance of the MRPs and the acceptance of recommendations made by clinical pharmacists	308 CKD patients	NR	Medication history interview, clinical and medication review by pharmacist. Recommendation were reported to the health care team	Not reported
Ohnishi et al. (2011) Japan [34]	Retrospective uncontrolled study (12 months)	Outpatient haemodialysis unit of a tertiary hospital	To explore the role of the pharmacists’ participation, we examined the influence of haemoglobin levels anteroposterior the participation	84 HD patients	62	Pharmacists provided drug information on renal anaemia to physicians, performed medication use evaluations based on laboratory data, proposed plans to change prescriptions based on medication use evaluations and provided drug information and lifestyle care point to patients	The counselling by pharmacists significantly decreased haemoglobin levels in the high group (12 g/dl) and significantly increased them in low group (10 g/dL)
Belaiche et al. (2012a) France [21]	Prospective uncontrolled study (6 months)	University hospital based nephrology clinic	To identify DRPs by a trained CP, their frequency and associated comorbidities	67 CKD patients	70	The CP interviewed patients and established a pharmacological profile, checked for drug–drug interactions, verified dose adaptation according to the last renal function tests and searched for self-medication and its potential nephrotoxicity. The pharmaceutical proposals were validated with the consulting nephrologist so as to optimise therapy during the following renal consultation	Not reported
Belaiche et al. (2012b) France [28]	Retrospective uncontrolled study (15 months)	Nephrology clinics of a university hospital	To assess the impact of clinical pharmacy services in outpatient nephrology clinics	42 CKD patients	64.9 (2.2)	Identification of DRPs by CP and documentation of recommendations	Not reported
Dashti-Khavidaki et al. (2012) Iran [23]	Prospective uncontrolled study (6 months)	Haemodialysis treatment centre of a teaching hospital	To assess the impact of clinical pharmacy services on the management of secondary complications in patients who were on HD, including bone metabolism disorders, anaemia and dyslipidaemia	86 HD	NR	CP reviewed patients medications and proposed modification according to laboratory data results to treating physicians	Serum Calcium was increased in hypocalcaemia patients and decreased in hypercalcaemia patients until it reached the optimal range in both groups A decline in serum Phosphate level was noted in hyperphosphataemia patients There was an increase and decrease in serum iPTH in suboptimal and supraoptimal range patients, respectively Haemoglobin concentration increased in anaemic patients and serum ferritin reached target values in all patients. Total cholesterol, low-density lipoprotein cholesterol and triglycerides decreased to near-optimal values in dyslipidaemia patients
Geerts et al. (2012) Netherlands [33]	Prospective uncontrolled study (unclear)	Primary health care	To assess the therapeutic advice formulated by pharmacists with help of a pharmacy medication alert system based on the renal function of patients aged ≥ 70 years with diabetes or cardiovascular disease	650 CKD patients	81 (6.7)	The pharmacists used a pharmacy medication alert system to assess the medication in relation to the reported eGFR and provided an alert for target drugs according to the Dutch guidelines for drug administration in reduced renal function	Not reported
Abu Ruz et al. (2013) Jordan [37]	Prospective uncontrolled study (3 months)	Nephrology ward of a general teaching hospital	To implement and evaluate the impact of pharmaceutical care service for hospitalised CKD patients in Jordan	130 CKD patients	56.3 (17.8)	The pharmacist Identified TRPs and interventions were discussed during ward rounds. Patients education and interview to improve patient adherence	17% of all TRPs were resolved, 5.5%were improved, and 37.4%were prevented through the clinical pharmacist interventions
Chen (2013) Singapore [25]	Prospective uncontrolled study (5 months)	Haemodialysis centre of a general hospital	To evaluate the prevalence of DRPs identified and the types of interventions made by MMS pharmacists	30 HD	62.3 (10.0)	Patients requested to bring their medication and see the pharmacist before the appointment with their physician. Pharmacist reviewed patients records, counsel the patients, identified and reported DRPs	Not reported
Jiang et al. (2013) Japan [38]	Prospective uncontrolled study (24 months)	Medical and surgical ICU of a university-affiliated hospital	To evaluate the benefits that may result from involving pharmacists in the care of septic patients receiving CRRT	144 Pre-intervention (71 patients) Post-intervention (73 patients) CRRT	Pre-intervention: 62.3 (17.0) Post-intervention: 57.9 (15.4)	Pharmacists completed 1 month of training before the study was started During the intervention period, the pharmacists assessed septic patients receiving CRRT daily and adjusted the dosage of antimicrobial drugs when needed. Recommendations were made to physicians and nurses at that time. All pharmacist recommendations were verbal and recorded on a specially designed pharmacist intervention form	Dosing adjustments were related to a reduced length of ICU stay from 10.7 ± 11.1 days to 7.7 ± 8.3 days (*p* = 0.037) in the intervention group, and to cost savings of 3525 USD (13,463 ± 12,045 vs. 9938 ± 8811, *p* = 0.038) per septic patient receiving CRRT in the ICU Suspected antimicrobial adverse drug events in the intervention group were significantly fewer than in the pre-intervention group (19 events vs. 8 events, *p* = 0.048) Dosing error events were significantly fewer in the post-intervention phase than in the pre-intervention phase (54 in 73 patients vs. 194 in 71 patients, *p* < 0.001)
Mousavi et al. (2013) Iran [22]	Retrospective/Prospective uncontrolled study (12 months)	University hospital based nephrology wards	To evaluate appropriateness of acid suppression therapy in kidney disease patients and to assess the role of clinical pharmacists to decrease inappropriate SUP prescribing and related costs for these patients	Pre-test phase (375 patients) Post-test phase (236 patients)	Pre-test phase 51.2 (18.3) Post-test phase 50.2 (18.8)	Pre-intervention phase: patient chart review by CP, develop SUP protocol, and provide educational sessions to doctors on SUP Post-intervention phase: Clinical pharmacists accompanied physicians on the ward rounds and advised on starting or stopping SUP	Not reported
Rani et al. (2013) India [50]	Prospective uncontrolled study (3 months)	Dialysis unit of a multispecialty university hospital	To assess the medication knowledge of CKD patients undergoing HD, to assess the effect of a CP provided continuous patient education in improving medication adherence and to evaluate the association between medication knowledge and medication adherence behaviour in HD patients	85 HD patients	50.52 (13.28)	Patient counselling and education (verbally and written). Patient interview to assess medication knowledge using MKAQ. To assess medication adherence pattern using BMQ	Not reported
Aberger et al. (2014) USA [41]	Prospective uncontrolled study (4 weeks)	Transplant clinic of a large urban hospital	To describes a telehealth system approach and preliminary results for the management of BP in renal transplant recipients and to enhance patient engagement and improve adherence to medications via a collaborative care, pharmacist-based, MTM program	66 Tx patietns	54	Telehealth system encompassing: home electronic BP monitoring designed to assess the efficacy of antihypertensive therapy. The pharmacist communicates BP reading data and dose modifications to the physician	Statistically significant reductions in average systolic and diastolic BP of 6.0 mm Hg and 3.0 mm Hg, respectively, at 30 days after enrolment (*p* < 0.01)
Arrabal-Durán et al. (2014) Spain [26]	Prospective uncontrolled study (10 months)	Hospital wards and emergency department of a general university hospital	To assess the characteristics of pharmaceutical interventions concerning the dose adjustment of these drugs in patients with CRF who are admitted into hospital	181 CKD patients	77.6 (12.5)	Medical history of each patient was reviewed by CP, recommendations for an adjustment were put in writing for the doctors	Not reported
Barnes et al. (2014) USA [27]	Retrospective uncontrolled study (12 months)	Primary care setting, Patient -Centred Medical Home associated with a major, academic health system	To increase the identification of CKD as a medical problem, increase the use of aspirin and ACEIs/ARBs in patients with CKD, and ensure that all medications prescribed to patients with CKD were dosed appropriately based on CG calculated CrCl	146 CKD patients	71.6 (12.2)	Review EMRs to identify CKD patients, review medication list, estimate CrCl and recommendations reporting to the physicians	Not reported
Gheewala et al. (2014) Australia [19]	Retrospective uncontrolled study (12 months)	Aged care facilities	To investigate the number and nature of DRPs identified and recommendations made by pharmacists in residents of aged care facilities To determine the extent of inappropriate prescribing of renally cleared medications in residents with CKD	847 CKD patients	84.9 (8.8)	DRPs identified, and recommendations made to resolve those DRPs by CP	Not reported
Holm et al. (2015) Norway [24]	Prospective uncontrolled study (6 months)	Internal medicine department of a general hospital	To describe the use of renal risk drugs in a population of patients with RI in an internal medicine department and investigate possible risk factors for such DRPs	79 CKD patients	78.7 (10.2)	The CP reviewed the patients’ drug regimen to classify DRPs related to renal function. DRPs identified were discussed with the physician	There was a significant correlation between the patients’ GFR and the number of DRPs, with an increasing number of DRPs with deteriorating renal function (*p* = 0.001, r = 0.371)
Pourrat et al. (2015) France [16]	Prospective uncontrolled study (7 months)	Community pharmacies	(1) To evaluate the ability of community pharmacists to identify drug related problems (DRP) in patients at risk for or suffering from renal impairment. (2) To evaluate the proportions of recommendations by CPs that lead to a modification by GP	177 CKD patients	78.1	The community pharmacist filled an electronic form for each prescription and verify whether the drug had to be adapted to renal function or was contraindicated Potential modification was proposed to the GP	Not reported
Venkateswararao et al. (2015) India [49]	Prospective uncontrolled study (6 months)	Dialysis unit of a teaching hospital	To evaluate the patient perception and degree of adherence to various treatment modalities (medication use, dialysis, life style modifications) by renal failure patients on HD To assess the effect of pharmacist’s interventions towards improving the adherence among the study population	58 HD patients	46.7 (13.3)	Patient counselling once in 2 weeks (total 3 sessions) was provided. Printed information leaflets and written information on dialysis note in regional language were provided to the patients. Adherence pattern before and after patient educational intervention was assessed	Not reported
Patricia and Foote (2016) USA [46]	Prospective uncontrolled study (17 months)	Regional dialysis units	To identify the extent and type of medication discrepancies and MRPs experienced by dialysis patients during pharmacist-initiated medication reviews and determine if the resulting recommendations made by the pharmacy team to the patient’s provider were accepted	90 HD	NR	Patients requested to bring their medication to dialysis unit and medication reconciliation conducted by the pharmacy team	Not reported
Ramadaniati et al. (2016) Indonesia [31]	Prospective uncontrolled study (3 months)	Medical wards and an ICCU in a major teaching hospital	To identify and evaluate drug-related problems (DRPs) in patients with CKD	105 CKD	NR	Identification of DRPs through the direct patient interview, discussion with nurses and assessment of patients’ medication charts and medical records	Not reported
Adibe et al. (2017) Nigeria [42]	Prospective uncontrolled study (5 months)	Nephrology units of three tertiary hospitals	To determine the prevalence of DTPs, identify the types of DTPs, and assess the outcomes of DTP interventions among renal patients receiving care in three Nigerian tertiary hospitals	287 patients with renal illnesses	72.34 (7.56)	Identify and report DRPs. Patient education and counselling	Not reported
Alshamrani et al. (2018) Saudi Arabia [59]	Retrospective uncontrolled study (3 months)	Outpatient haemodialysis unit of a tertiary hospital	To determine the prevalence of polypharmacy and the Medication Related Problems in haemodialysis patients	83 HD patients	Median age 63, IQR (49–1)	The pharmacy resident reviewed electronic medical records and analysed each medication regimen for eligible patients to identify MRPs	Not reported
Chandrasekhar et al. (2018) India [60]	Prospective interventional study (12 months)	Outpatient nephrology department	To evaluate medication adherence behaviour of patients using questionnaire and enhance adherence by various cost effective interventions which have greater effect on the health of patients with CKD	163 CKD patients	–	Patient counselling by pharmacist and patient information leaflet was carried out using a proper management plan and with the help of physician and feedback information was collected	Not reported
Imamura et al. (2018) Japan [61]	Retrospective uncontrolled study (unclear)	Hospital	To determine whether multidisciplinary care could help prevent worsening renal function associated with CKD	150 CKD patients	72.3 (10.5)	The multidisciplinary care was provided by a team of nephrologists, diabetologist, nurses, diabetes educator, dietitians and pharmacists	The eGFR significantly improved between before and after multidisciplinary care from − 5.46 to − 0.56 mL/min/1.73 m^2^/year, respectively Values for uric acid, LDL, and HbA1c were significantly reduced among patients with improved eGFR

*ACEi* angiotensin converting enzyme inhibitors, *ACR* albumin:creatinine ratio, *ARBs* angiotensin receptor blockers, *BMQ* Brief medication questionnaire, *BP* blood pressure, *CrCl* creatinine clearance, *CG* Cockcroft-Gault, *CKD* chronic kidney disease, *CP* clinical pharmacist, *CRF* chronic renal failure, *CRRT* Continuous renal replacement therapy, *DRPs* drug related problems, *eGFR* estimated glomerular filtration rate, *EMRs* electronic medical records, *FBC* full blood count, *GFR* glomerular filtration rate, *GP* general practitioner, *HbA1c* glycosylated haemoglobin, *HD* haemodialysis, *ICCU* intensive critical care unit, *ICU* intensive care unit, *iPTH* intact parathyroid hormone, *IQR* interquartile range, *MKAQ* Medication knowledge assessment questionnaire, *MMS* medication management service, *MRPs* medication related problems, *MTM* medication therapy management, *NR* not reported, *RI* renal impairment, *SUP* stress ulcer prophylaxis, *TRPs* therapy related problems, *Tx* transplantation

### Study characteristics

The 47 studies were carried out in a variety of geographic locations: USA (n = 10), Iran (n = 5), India (n = 7), France (n = 3), Spain (n = 3), Jordan (n = 2), China (n = 2), Japan (n = 3), Singapore (n = 2), Nigeria, Taiwan, Australia, Saudi Arabia, Germany, Netherlands, Indonesia, Norway, Canada and the UK (n = 1 in each country). Two studies from 2008 to 2009 were not included in the systematic review of Salgado et al. [7], hence were considered as part of this review. Thirty-one studies were conducted in hospital settings (wards, intensive care units (ICU), clinics, departments and dialysis units) and 16 in primary care settings, including clinics and community pharmacies. The follow-up time in all included papers ranged from 4 weeks to 24 months with a mean of 9.4 (standard deviation, SD = 5.08) months, with four studies with unclear duration.

The majority of studies (n = 27) used an uncontrolled study design, 21 prospective and six retrospective. The remaining 20 were controlled, ten of which were randomised and ten non-randomised. According to Thomson Reuters Journal Citation Report at the time of publication the median impact factor of the journals of articles included was 1.348 (IQR 0.52–2.01), n = 45, two journals did not have an impact factor at the time of publication.

Patient mean age was 46.7–84.9 years, with five studies failing to report age [23, 29, 31, 46, 60]. Of the total of 11,122 patients from all studies, 9151 were at various stages of chronic kidney disease not on dialysis, 1036 were haemodialysis (HD) dependent, 533 receiving other forms of renal replacement therapies such as continuous renal replacement therapy (CRRT) or continuous veno-venous hemofiltration (CVVH), and 402 were transplant patients.

Outcomes were reported in 37 papers, with 25 of these (67.6%) also reporting details of the processes of care, and four (10.8%) reporting structures, processes and outcomes. Outcomes reported were: clinical only (17, 45.9%), economic with linked clinical (5, 13.5%), humanistic with linked clinical (4, 10.8%), humanistic only (2, 5.4%) and economic only (2, 5.4%). The 10 remaining papers did not report outcomes measures with one (2.1%) that reported structure and process indicators only and 9 (19.1%) reported process indicators only.

### Resources for care provision: structures

Structures were poorly reported in all studies, with only two giving some details of multidisciplinary team involvement [52, 61], while, none on the pharmacist skill mix or time allocation. The only aspect of structures reported relating to training which was given in five studies. In one, pharmacists and pharmacy residents were engaged in a two-week training of literature review and patient assessments [35]. A community pharmacist based study described a workshop covering clinical presentations of CKD, managing drug-related problems and discussing patient cases [48]. Similar training was described for community pharmacists, [18] and hospital clinical pharmacists [16], to enable them to identify patients with renal insufficiency and perform dose adjustments. A four session course to all members of the multidisciplinary team prior to the study was described in one article [61].

### Characteristics of clinical pharmacy practice: processes

All studies provided some description of the processes undertaken by the pharmacists, although the detail provided varied considerably and was generally lacking. The majority of processes (often labelled as interventions) included medication chart review to identify any drug-related problems (DRPs) [15–31]. Many studies reported pharmacists’ interventions in: modifying drug doses and recommending new pharmacotherapy; [16, 19, 21–23, 25–27, 29, 30, 32–40, 52, 59]; interacting with a member of the multidisciplinary team; [15–17, 19–21, 23–25, 27, 31, 32, 34–38, 40–43] requesting and monitoring laboratory parameters; [15, 23, 25, 27, 33, 34, 36, 37, 43] assessing appropriateness of medications prescribed for hospitalised patients at each point of care; [17, 22, 29, 30, 35–38, 40, 57]. Fewer studies described pharmacist processes at out-patient, pharmacist-led clinics relating to the management of specific CKD complications, such as anaemia; [34, 39, 44] hypertension and diabetes; [54] managing hypertension through telemedicine; [41] optimising dyslipidaemia management; [37, 45] improving haemoglobin A1c levels (HbA1c); [43] and emphasising smoking cessation. [37, 43] Development of protocols and compiling and updating guidelines were also described in two studies [22, 34]. Performing medication reconciliation [46]; providing patient medication counselling, education on disease status or medication, conducting motivational interviews to improve adherence were also reported [15, 25, 27, 29, 30, 34, 36, 37, 42, 43, 47–50, 55, 57, 58, 60]. A number of studies reported pharmacists’ participation in ward rounds [17, 22, 35, 37, 38, 40], providing educational sessions to healthcare professionals [22, 34] and performing activities such as medication use evaluations [34]. There were no reports of pharmacist prescribing activities; one study described the process of deprescribing to optimise medication use [59].

Fewer studies provided any data on time spent on specific activities. Interaction time between pharmacist and patients were reported in two studies, varying from 15 to 30 min [43, 50] and the timeframe in which the services were provided ranged from daily [35–38, 40] to every three months [47].

Across all studies, the pharmacists identified 5302 drug-related problems in 2933 patients. Pharmacists made 3160 recommendations to healthcare professionals with an acceptance rate varying from 33.3% in a community setting; [16] 46.43% in a dialysis unit; [59] to around 95% in hospital settings [17, 24, 42, 51, 52, 57]. Only three studies reported the clinical significance of recommendations. Of these 26% were of moderate to [29], 48.8% of major clinical significance [51] and 47% serious severity [20].

A pharmacist-based quality improvement programme consisting of pharmacists’ interactions with the patients and electronic collaboration with the physicians was associated with a significant improvement in the measurement of PTH during the study period [15]. Pharmacists’ interventions led to medication therapy modifications [16–21, 24–29, 31, 33, 37, 42, 46] and resolving medication record discrepancies [46, 57]. Patients’ compliance with ongoing blood pressure (BP) monitoring post kidney transplantation was significantly improved with pharmacists’ input [41]. Counselling by pharmacists significantly improved medication adherence in patients with CKD [47, 50, 60].

### Clinical outcomes

The final column of Tables 1 and 2 titled ‘Main outcomes achieved’ provides a detailed summary of main results and statistical significance values related to each of the studies summarised below. Clinical outcomes only were reported in (n = 17) studies. A pharmacist-based quality improvement programme in a pragmatic randomised controlled study reported that patients in the intervention arm were prescribed more classes of antihypertensive medications than those in the control arm [15]. In a 6-month cluster randomised trial, pharmacists attending a structured training and communication-network programme (ProFil) and managing hypertension in CKD patients demonstrated larger reduction in systolic blood pressure (BP) of the intervention group compared to the usual care group [48].

Intervention in the management of BP in CKD and haemodialysis resulted in achieving target BP in the intervention versus the control group [32, 54, 55], significant reductions in mean systolic and diastolic BP in a group of kidney transplant recipients [41], and significant reduction in systolic and diastolic BP in diabetic nephropathy [43]. Only one article showed that pharmacists’ intervention in an intensive care unit (ICU) setting reduced the length of ICU stay [38]. Another study reported reduction in the length of stay in the intervention group by 1.3 days (*p* < 0.001) and reduced unplanned admission by 27% (*p* = 0.047) [52]. One further study showed no difference of pharmacists’ intervention compared to usual care on hospital readmission outcomes [57]. Pharmacists were also involved in the monitoring of kidney function in patients with CKD and demonstrated significant differences in measuring CrCl between discharge and admission [36]. However, one study demonstrated no difference in the mean serum creatinine or estimated glomerular filtration rate (eGFR) between the intervention and control groups [58]. A retrospective controlled study reported improvement in eGFR, uric acid, cholesterol and HbA1c in the intervention group compared to the control group after multidisciplinary care, however, pharmacists’ contribution to the care was not clearly reported [61].

Four studies gave outcomes of pharmacists managing anaemia in CKD patients [34, 39, 44, 55], with significant haemoglobin values within target range in pharmacist-led clinic. Time to achieve target haemoglobin was 28 days in the pharmacist-managed group compared with 41 days in the usual care group [44]. While the proportion of patients achieving target haemoglobin was not significant, pharmacist intervention significantly improved haemoglobin and iron monitoring by improving compliance to therapy [44]. Pharmacist counselling significantly improved haemoglobin levels in one study [34], with haemoglobin concentration and Transferrin saturation (TSAT%) increasing significantly and serum ferritin reaching target values in a prospective uncontrolled study [23].

An uncontrolled study of the impact of on managing secondary complications of haemodialysis patients resulted in significantly increased median serum calcium in those with hypocalcaemia and decreased values in hypercalcaemia, a decline in serum phosphate in patients with hyperphosphataemia, and an increase and decrease in serum iPTH in patients with sub-optimal and supra-optimal levels respectively [23].

Pharmacists’ interventions in a pragmatic, cluster randomised study improved screening of proteinuria between an interventions compared to control group [45]. A non-randomised controlled study of pharmacist involvement in a monitoring program for CKD reported significant differences in CrCl between discharge and admission in both the control and intervention groups [36].

### Humanistic outcomes

In a cluster, randomised study health related quality of life (HRQoL) improved significantly compared to control in a group of haemodialysis patients receiving pharmacist intervention over a 6-month period [30]. In a non-randomised controlled study, HRQoL domains were not significantly impacted by the additional pharmacist care in kidney transplants [47]. A multicentre RCT reported significant improvement in HRQoL scores in the intervention group compared to control [53].

Patient satisfaction reported in two randomised controlled studies: 92% of patients had positive feelings about pharmacists’ involvement in their care and felt that the pharmacist provided beneficial information [15] and 43% of patients were ‘very satisfied’ with the care received and were willing to receive future care from the pharmacist [45]. A cross-sectional prospective study demonstrated that patients were greatly satisfied with the intervention [58].

### Economic outcomes

Only seven studies reported economic outcomes resulting from pharmacist input [22, 35, 38–40, 44, 56]. One study reported that pharmacists in the ICU could contribute to significant cost savings in septic patients, with antimicrobial prescribing efficiencies accounted for 34.7% of total savings [38]. In a study investigating an ICU pharmacist dosing adjustment programme, the mean ICU hospitalisation costs per patient decreased significantly with total savings of 2669.5 USD per patient [40]. Jiang et al. demonstrated that pharmacist dosing adjustment resulted in drug cost savings per patient of 2345.98 USD with antibiotics accounting for 64.5% of all cost savings. The presence of an ICU pharmacist resulted in 2346 USD savings per patient receiving continuous renal replacement therapy [35]. Debenito et al. reported that the mean weekly dose of erythropoiesis-stimulating agents (ESAs) was significantly less in the pharmacist-managed group than the usual care group and the annualised ESA cost per patient reduced by 1288 USD [44], whereas, Aspinall et al. reported lower average dose of darbepoetin in the pharmacist-managed ESA clinic compared to the usual care [39]. Mousavi et al. showed that the cost per patient for inappropriate stress ulcer prophylaxis administration in patients with insufficient renal function was reduced by pharmacists’ intervention [22]. A multicentre RCT reported that pharmaceutical care costed more per quality adjusted life year (QALY) gained compared to usual care [56].

## Discussion

There are a number of important key findings that have arisen from this review and these are outlined below. Forty-seven new studies have been published in the intervening 8 year period since a previous similar review [7]. Ten of these are of a ‘gold standard’ RCT design and the quality of the controlled studies included is generally poor. Structures and processes were very poorly reported and none of the studies included consideration of pharmacist prescribing—which is considered in several countries, where it has been implemented, to be a significant advance in pharmacy practice. The process indicators in the original review [7] and this review were very similar but this review identified papers with clear shift from only identifying drug-related problems to more involvement of the pharmacist in medication therapy management. Most of the studies in this review continue to focus on and report details of DRPs as an indicator of the process of pharmacy practice. Some of these considered the clinical significance of these DRPs but this was not universal. Less focus on clinical, humanistic and economic outcomes was observed in majority of the papers in both reviews.

Many of the uncontrolled studies had a variety of quality deficiencies including; lack of comprehensive explanation of the pharmacists’ intervention, under-reporting of adverse events and insufficient information to allow reproduction of the study for interested readers. Few studies lacked some important information leading to poor scoring of the study, such as lack of clarity in stating the study aim, [35] the number of participants, the population from where the sample was drawn, duration of the data collection or the study period, frequency of follow-up, and some studies were unable to clearly state the distribution of the confounders in both groups [15, 22, 30, 35, 39, 45].

The majority of the 20 controlled studies were of ‘fair’ quality with the exception of four that were considered ‘weak’ [22, 55, 56, 58]. High quality RCTs with low levels of bias generate the highest level of evidence [62]. However, the availability of quality evidence in this area is limited with only 5 RCTs were included in this review and 4 in a previous review by Salgado et al. [7]. The RCTs in both reviews lacked sufficient information on the randomisation process, in addition to poor detail on any blinding process of the care-giver and the care-receiver (however, it might be a challenge to blind in some study designs) so jeopardising the quality of these studies [63]. It is therefore evident that there has been a limited amount of high quality research published for the benefits of clinical pharmacy practice in CKD. There is particularly a paucity of evidence from RCTs offering a robust evidence base for practice. Despite this criticism there is a growing body of information in relation to some aspects of clinical pharmacy practice that offers some insights to the developing quality of services provided making real and significant differences to the outcomes of patients. This, however, needs to be verified through even more robust RCTs that are better resourced, designed and executed.

The gathering of more gold standard evidence such as RCTs is essential to enable measuring the impact of clinical pharmacists’ intervention in patients with CKD compared to standard care. Furthermore, there is an identified need to carry out studies with explicit details and accurate definitions including the setting, the participants, the randomisation process and the interventions of interest.

It is of paramount importance that detailed descriptions of the interventions, in terms of structures and processes and outcomes, are included in publications to allow them to be reproduced and for readers to consider the studies within the context of their own practice [64]. Most papers lacked sufficient details of the clinical pharmacy practices so making it difficult to fully understand the activity. Without full insight to practice it is difficult to fully understand the context and characteristics of practice and so reproduce the structures and processes in wider settings. This is not just a deficiency of studies in CKD since a study by Schroter et al. to assess the replicability of published clinical interventions, in a variety of clinical settings, reported that 57% of the studies had insufficient description of the intervention of interest to make it replicable [65]. A tool produced by Correr et al. to address the lack of intervention descriptions in clinical pharmacy research (Descriptive Elements of Pharmacist Intervention Characterization Tool) DEPICT is a validated instrument for accurately describing the details of pharmacist interventions performed as part of clinical pharmacy practice [66]. This tool could be used as a guidance to structurally describe the intervention of interest in pharmacy practice research.

Additionally it should be noted that in CKD there are no studies that have specifically investigated prescribing as part of clinical pharmacy practice and there are no full description of structure, processes and outcomes as they relate to prescribing practice. A systematic review by Tesfaye et al. published in 2017 of the prevalence of inappropriate prescribing and the impact of pharmacists’ interventions reported significant reduction in inappropriate prescribing when physicians received immediate concurrent feedback from a clinical pharmacist [67]. The review showed minimal involvement of the pharmacist in the role of prescribing for patients with CKD. Despite the increased recognition of prescribing models such as independent, supplementary or collaborative [6], there was limited published evidence to lead to the best practice model for prescribing.

There is also a need to stimulate more of a research culture within clinical pharmacy practice. A paper by Peterson et al. reported that lack of time, lack of opportunities, lack of training and never being asked to participate in a research were major barriers for pharmacists’ engagement in research [68]. A systematic review by Awaisu et al. concluded that pharmacists are aware of the value of research to enable them advance pharmacy practice and indicate their willingness to be involved in independent research and in practice-based research networks. However, lack of time, training and support were the main barriers [69].

A strength for this review is that the protocol was peer reviewed and registered with PROSPERO. The protocol was devised in accordance with PRISMA-P (Preferred Reporting Items for Systematic review and Meta-Analysis Protocols) standards [9] and the systematic review was conducted and reported in accordance with PRISMA (Preferred Reporting Items for Systematic Review and Meta-Analysis) standards [10]. In terms of limitations, publication bias could potentially affect the selecting process of the articles, since no study was identified to show the negative impact of clinical pharmacy services in caring for patients with CKD. One further limitation is the exclusion of papers in languages other than English potentially leading to the omission of relevant papers.

In conducting RCTs, it has been recognised that it is vital to be careful in the selection and recording of outcomes to build up a coherent dataset [70–73]. Moreover, consistency in the use of outcomes will aid future users of the services and those involved in resource allocation, planning and implementation of clinical pharmacy services [72]. It is evident from this review that where RCTs were conducted, there was no consistency in the selection and reporting of outcomes. These issues could be addressed with the development and application of agreed standardised sets of outcomes [73]. Research on core outcome set definitions for clinical pharmacy practice is ongoing in many areas such as polypharmacy [74] but this appears to be lacking in CKD, which could be a potential area of work in the future.

## Conclusion

There is some evidence for the outcomes of pharmacists’ intervention in patients with CKD but this is generally of low quality and insufficient volume. The controlled studies in this systematic review showed that pharmacist interventions improved patients’ clinical outcomes such as Hb levels, CrCl, PTH and calcium levels. However, these studies lacked detail on reporting of the humanistic outcomes and there remains a paucity of evidence demonstrating economic impact of pharmacists’ interventions.

There is some evidence since the last review that shows positive contributions of pharmacists’ involvement in the multidisciplinary team to provide care to patients with CKD. This includes evidence on the structure, processes of care and the outcomes of pharmacists’ intervention in patients with CKD. More high-quality research in this area is warranted.

## Electronic supplementary material

Below is the link to the electronic supplementary material.

Supplementary material 1 (DOCX 21 kb)
