# Supplementary material for: Clinical pharmacy practice in the care of Chronic Kidney Disease patients: a systematic review
Source: Int J Clin Pharm. 2019 Apr 9;41(3):630–66. doi: 10.1007/s11096-019-00816-4 (PMC6554252; doi:10.1007/s11096-019-00816-4)
Supplement: Supplementary file 1 — Supplementary material 1 (DOCX 21 kb) [file 11096_2019_816_MOESM1_ESM.docx]

**Clinical pharmacy practice in the care of Chronic Kidney Disease patients: a systematic review**

**Submission: IJCP- IJCP-D-18-00675R1**

**Supplementary Materials**

**Quality Assessment Tables**

1. Quality assessment tool for quantitative Randomized Controlled Trials (MMAT)

|  | Santschi et al. 2011 | Dashti-Khavidaki et al. 2013 | Cooney et al. 2015 | Chang et al. 2016 | Qudah et al. 2016 | Mateti et al. 2017 | Mateti et al. 2018 a | Mateti et al. 2018 b | Anderegg et al. 2018 | Tuttle et al. 2018 |
| --- | --- | --- | --- | --- | --- | --- | --- | --- | --- | --- |
| 1. Are there clear quantitative research questions or objectives? | Can’t tell | Yes | Yes | Yes | Yes | Can’t tell | Can’t tell | Can’t tell | Yes | Yes |
| 2. Do the collected data allow address the research question or objectives? | Yes | Yes | Yes | Yes | Yes | Yes | Yes | Yes | Yes | No |
| 3. Is there a clear description of the randomization? | No | Can’t tell | Yes | Yes | Can’t tell | Yes | No | Yes | No | Yes |
| 4. Is there a clear description of the allocation concealment (or blinding when applicable)? | No | No | Yes | No | Yes | Yes | No | No | No | Yes |
| 5. Are there complete outcome data (80% or above)? | Yes | No | No | Can’t tell | Yes | No | Can’t tell | Can’t tell | Can’t tell | Yes |
| 6. Is there low withdrawal/drop-out (below 20%)? | Yes | No | Yes | No | Yes | No | No | No | Can’t tell | Yes |

Key: Yes: criteria met, No: criteria not met, Can’t tell: no enough information provided to make a decision and NA: Not Applicable

1. Quality assessment tool for quantitative non-randomized studies

|  | Aspinall et al. 2012 | Mousavi et al. 2013 | Via-Sosa et al. 2013 | Jiang et al. 2013 | Jiang et al. 2014a | Jiang et al. 2014b | Cabello-Muriel et al. 2014 | Debenito et al. 2014 | Joost et al. 2014 | Staino et al. 2015 | Venkateswararao et al. 2015 | Chia et al. 2017 | Xu et al. 2018 |
| --- | --- | --- | --- | --- | --- | --- | --- | --- | --- | --- | --- | --- | --- |
| 1. Are there clear quantitative research questions or objectives? | Yes | Yes | Yes | Yes | Yes | Yes | Yes | Yes | Yes | Yes | Yes | Yes | Yes |
| 2. Do the collected data allow address the research question or objectives? | Yes | Yes | Yes | Yes | Yes | Yes | Yes | Yes | Yes | Yes | Yes | Yes | No |
| 3. Are participants (organizations) recruited in a way that minimizes selection bias? | Can’t tell | Yes | Yes | Yes | Can’t tell | Yes | Yes | Can’t tell | Yes | Can’t tell | Can’t tell | Can’t tell | Can’t tell |
| 4. Are measurements appropriate regarding the exposure/intervention and outcomes? | Yes | Yes | Yes | Yes | Yes | Yes | Can’t tell | Yes | Yes | Yes | Can’t tell | Yes | Yes |
| 5. In the groups being compared, are the participants comparable? | No | Can’t tell | Yes | Yes | Yes | Yes | Can’t tell | Yes | Yes | No | Can’t tell | Yes | Yes |
| 6. Are there complete outcome data (80% or above), or acceptable response rate (60% or above)? | Yes | Yes | Yes | Yes | Yes | Yes | Yes | Yes | Yes | NA | Yes | Yes | Yes |

Key: Yes: criteria met, No: criteria not met, Can’t tell: no enough information provided to make a decision and NA: Not Applicable

1. Quality assessment tool for quantitative non-randomized studies

|  | Kelly & Booth 2008 | Dashti-Khavidaki et al. 2009 | Vessal 2010 | Castelino et al. 2011 | Ohnishi et al. 2011 | Belaiche et al.) 2012a | Belaiche et al. 2012b |
| --- | --- | --- | --- | --- | --- | --- | --- |
| 1. Are there clear quantitative research questions or objectives? | No | Yes | Can’t tell | Yes | Can’t tell | Can’t tell | Can’t tell |
| 2. Do the collected data allow address the research question or objectives? | Can’t tell | Can’t tell | Yes | Yes | Yes | Yes | Yes |
| 3. Is the sampling strategy relevant to address the quantitative research question? | Can’t tell | Can’t tell | No | Yes | Yes | Yes | Yes |
| 4. Is the sample representative of the population understudy? | Can’t tell | Can’t tell | Can’t tell | Yes | Yes | Yes | Yes |
| 5. Are measurements appropriate (clear origin, or validity known, or standard instrument)? | Yes | Can’t tell | NA | Yes | Yes | Can’t tell | Yes |
| 6. Is there an acceptable response rate (60% or above)? | NA | NA | Yes | NA | Yes | Yes | NA |

Key: Yes: criteria met, No: criteria not met, Can’t tell: no enough information provided to make a decision and NA: Not Applicable

1. Quality assessment tool for quantitative descriptive studies (continued)

|  | Dashti-Khavidaki et al. 2012 | Geerts et al. 2012 | Abu Ruz et al. 2013 | Chen et al. 2013 | Rani et al. 2013 | Aberger et al. 2014 | Arrabal-Duran et al. 2014 | Barnes et al. 2014 |
| --- | --- | --- | --- | --- | --- | --- | --- | --- |
| 1. Are there clear quantitative research questions or objectives? | Yes | Yes | Yes | Yes | Yes | Can’t tell | Yes | Yes |
| 2. Do the collected data allow address the research question or objectives? | Yes | Can’t tell | Yes | Can’t tell | Yes | Yes | Yes | Yes |
| 3. Is the sampling strategy relevant to address the quantitative research question? | Can’t tell | Yes | Yes | Can’t tell | Can’t tell | Yes | Yes | Yes |
| 4. Is the sample representative of the population understudy? | Can’t tell | Yes | Yes | Can’t tell | Yes | Can’t tell | Yes | Yes |
| 5. Are measurements appropriate (clear origin, or validity known, or standard instrument)? | Yes | NA | Yes | No | Can’t tell | Yes | Yes | Yes |
| 6. Is there an acceptable response rate (60% or above)? | NA | Yes | Yes | NA | Yes | Yes | NA | Yes |

Key: Yes: criteria met, No: criteria not met, Can’t tell: no enough information provided to make a decision and NA: Not Applicable

1. Quality assessment tool for quantitative descriptive studies (continued)

|  | Gheewala et al. 2014 | Holm et al. 2015 | Pourrat et al. 2015 | Patricia & Foote 2016 | Ramadaniati et al. 2016 | Adibe et al. 2017 | Alshamrani et al. 2018 | Imamura et al. 2018 | Chandrasekhar et al. 2018 |
| --- | --- | --- | --- | --- | --- | --- | --- | --- | --- |
| 1. Are there clear quantitative research questions or objectives? | Yes | Can’t tell | Yes | Yes | Yes | Yes | Yes | Yes | No |
| 2. Do the collected data allow address the research question or objectives? | Yes | Can’t tell | Yes | Can’t tell | Yes | Yes | Yes | Yes | Yes |
| 3. Is the sampling strategy relevant to address the quantitative research question? | Yes | Yes | Can’t tell | Can’t tell | Yes | Yes | Yes | Yes | Yes |
| 4. Is the sample representative of the population understudy? | Yes | Can’t tell | Yes | Can’t tell | Yes | Yes | Can’t tell | Yes | Yes |
| 5. Are measurements appropriate (clear origin, or validity known, or standard instrument)? | Yes | Yes | NA | Yes | Yes | Yes | Yes | Yes | Can’t tell |
| 6. Is there an acceptable response rate (60% or above)? | NA | NA | Yes | NA | NA | Yes | Yes | Yes | Yes |

Key: Yes: criteria met, No: criteria not met, Can’t tell: no enough information provided to make a decision and NA: Not Applicable
